# How to engineer the unknown: Advancing a quantitative and predictive understanding of plant and soil biology to address climate change

**DOI:** 10.1371/journal.pbio.3002190

**Published:** 2023-07-17

**Authors:** Simon Alamos, Patrick M. Shih

**Affiliations:** 1 Department of Plant and Microbial Biology, University of California, Berkeley, Berkeley, California, United States of America; 2 Feedstocks Division, Joint BioEnergy Institute, Emeryville, California, United States of America; 3 Environmental Genomics and Systems Biology Division, Lawrence Berkeley National Laboratory, Berkeley, California, United States of America; 4 Innovative Genomics Institute, University of California, Berkeley, Berkeley, California, United States of America; University of California, Davis, UNITED STATES

## Abstract

Our basic understanding of carbon cycling in the biosphere remains qualitative and incomplete, precluding our ability to effectively engineer novel solutions to climate change. How can we attempt to engineer the unknown? This challenge has been faced before in plant biology, providing a roadmap to guide future efforts. We use examples from over a century of photosynthesis research to illustrate the key principles that will set future plant engineering on a solid footing, namely, an effort to identify the key control variables, quantify the effects of systematically tuning these variables, and use theory to account for these observations. The main contributions of plant synthetic biology will stem not from delivering desired genotypes but from enabling the kind of predictive understanding necessary to rationally design these genotypes in the first place. Only then will synthetic plant biology be able to live up to its promise.

## Abstract 2

How can we attempt to engineer what we do not fully understand? This Essay argues that main contributions of plant synthetic biology in addressing climate change will stem not from delivering desired genotypes but from enabling the kind of predictive understanding needed to rationally design these genotypes in the first place.

## The promise and engineering challenge of using plants to sequester carbon in the soil

The accumulation of greenhouse gasses in the atmosphere and its consequences for climate patterns threaten our current way of life. At the same time, the phenomena that underlie these trends are of such breathtaking complexity that scientists are hardly equipped to fully understand—not to mention engineer—them. This disparity between what is needed and what is possible has inspired ambitious private and public “moonshot” initiatives that promise a number of panaceas for sequestering atmospheric carbon fixed by photosynthesis. A high profile example of such initiatives is the engineering of plants to stash away fixed carbon in the soil.

Soils contain 3 times as much carbon as the atmosphere and more carbon than the atmosphere and vegetation combined [1,2]. Thus, in principle, even a modest increase in soil carbon could draw a substantial amount of CO_2_ out of the atmosphere. Soil carbon exists mostly in the form of dead organic matter in different states of decomposition in the top 30 cm and comes primarily from carbon fixed by photosynthesis and deposited from plant roots (as exudates (Box 1), mucilage, or decaying biomass, reviewed by [2–5]). In this sense, one could argue that the technology to pump atmospheric carbon into the soil has existed for hundreds of millions of years, since the first plants colonized land. Release of carbon from the soil is controlled by the oxidation of these organic molecules by microbial respiration. Soil organic matter is usually conceptualized as 2 or more pools with different rates of decay: “ephemeral” pools, which are respired with a rate similar to that of biomass deposition (on the order of weeks to months), and “lingering” pools, which persist for a much longer time (decades or even centuries). Thus, it is the existence of this longer-lived pool that allows for the accumulation of carbon in the soil. Because agricultural practices during the past few centuries have led to the loss of substantial amounts of soil carbon, modern soils are far from “at capacity” in terms of carbon storage [1,2]. Thus, it is rationalized that it should be possible to partially revert the past century’s losses in soil carbon by increasing the rate of growth of the lingering pool. This could be achieved by technologies that increase the rate with which plant biomass enters the persisting soil carbon pool or reduce the rate with which this pool is degraded [1,6,7].

**Box 1. Glossary**

**Root exudates**

Substances released into the surrounding medium by plant roots. Typically a mixture of soluble organic compounds such as proteins and sugars.

**Synthetic biology**

Engineering discipline whose substrate is the genetic makeup of living cells and organisms.

**C3 carbon fixation**

Metabolic pathway found in most land plants that transforms 3 molecules of CO_2_ into a 3-carbon molecule using light energy.

**Auxin**

Mobile plant hormone whose distribution dictates cell expansion and division in the root, among multiple other aspects of plant growth and development.

**Harvest index**

Fraction of the total biomass energy that goes into the harvested portion of a crop.

These technologies have garnered substantial attention in popular media articles aimed at the scientifically curious public, as well as in technical reports for researchers and policy makers. As a result, recent years have seen a significant influx of funding that has fertilized a burgeoning startup ecosystem dedicated to plant-based soil carbon sequestration technologies. Yet, both critics and candid advocates recognize that this vision rests on untested or even faulty assumptions regarding the mechanisms and dynamics of carbon cycling in the soil [2,7–12]. Even obviating this key missing understanding, the success of this program depends to a large degree on our capacity to rationally engineer complex multicellular organisms like plants and their interactions with what is arguably the most complex ecological environment in nature, namely, the soil ecosystem. Indeed, the biological substrate at our disposal for plant engineering has already been functionalized through eons of evolutionary history following principles that we have only begun to grasp. Thus, our complete naïveté of the complexity of cells thwarts most rational engineering efforts. Before we have had time to even begin to learn to effectively engineer the cell, many have proposed we expand towards engineering not just whole organisms, but whole ecosystems. How can we possibly hope to engineer the unknown?

We hope to show that engineering goals that seem intractably complex can be gradually and organically achieved if the focus is kept on building a quantitative and predictive understanding of the system at hand. Any engineering field must be able to predict with precision the outcome of future experiments by combining empiricism with theoretical modeling. Further, a mature engineering discipline will have the tools at its disposal to test these predictions. We are hardly alone in this view. The recent White House executive order to advance biotechnology lists as a key research area the “predictive engineering of complex biological systems, including the designing, building, testing, and modeling of entire living cells, cell components, or cellular systems; quantitative and theory-driven multidisciplinary research to maximize convergence with other enabling technologies” [13]. To illustrate this view in the context of engineering plants for climate change, we structure our discussion around a comparison of 2 case studies. First, we walk through the past scientific advances that enabled recent successes in engineering photosynthesis. Using this historical perspective, we show that such engineering successes can be ascribed to the kind of quantitative and predictive understanding pursued early on in the field. For our second case study, we shift our focus from the past to the future. We critically assess the missing knowledge gaps and prospects of genetically engineering plants for soil carbon sequestration, a fairly new approach that combines an ambitious engineering goal and a potentially large-scale impact. Specifically, we use the ability to predict the phenotype of yet unseen genotypes as a yardstick with which to measure the maturity of this technology. Finally, we propose synthetic biology (Box 1) applications that could be used to achieve this kind of predictive foundation.

### Case study 1: Engineering photosynthesis

Arguably, the most successful examples of rational genetic engineering in plants to date are examples of engineering photosynthesis. We focus specifically on recent engineering efforts to enhance light harvesting efficiency pursued by the Realizing Increased Photosynthetic Efficiency (RIPE) project. Remarkably, this effort can be ascribed to a continuous dialog between quantitative measurements, theory, and experiments going back to a string of theoretical papers by Xin-Guang Zhu, Steve Long, and others. By integrating all known quantitative and theoretical knowledge about C3 carbon fixation (Box 1) and crop physiology, the authors were able to pinpoint precise engineering targets and predict their effect in terms of crop yield. In fact, this is but one of multiple promising threads in the rational engineering of photosynthesis light and carbon reactions [14,15]. This kind of predictive and rational engineering is not readily found in other areas of plant biology. How did photosynthesis research as a field arrive at this kind of understanding? The question is worth asking today because, with the benefit of hindsight, we can learn lessons that can be applied to newer problems in plant biology such as engineering plants for increasing the carbon content of soils. We argue that these successes are the result of 3 central goals pursued from the very inception of the field, namely: identifying the key control variables and characterizing their quantitative effects, accounting for observations using mechanistically inspired theoretical models, and testing model predictions using genetic perturbations.

### Identifying the key control variables and characterizing their quantitative effects

**Fig 1:**
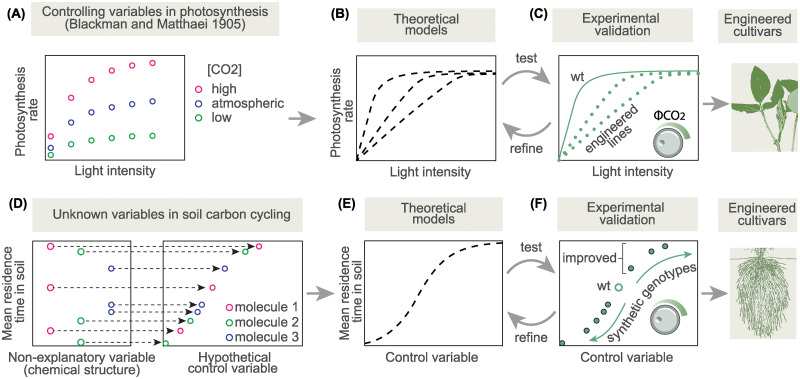
Engineering advances as the natural consequence of striving for predictive understanding. **(A)** The first step toward a predictive understanding is pinpointing the key control variables and how their input quantitatively defines the output phenotype. In the case of C3 photosynthesis, pioneering work by Blackman and Matthaei around the turn of the century described the key variables and their functional relationship to the rate of photosynthesis. **(B)** The observations described in (A) were progressively accounted for using mathematical models in dialog with experiments. **(C)** Experiments such as genetic perturbations were used to validate and refine theoretical predictions. For example, lines were engineered to switch back and forth the slope of the green curves with different speeds, which was predicted to increase carbon fixation under fluctuation light regimes. This predictive understanding enabled the engineering of cultivars with improved photosynthesis. **(D)** Left: Large uncertainties reveal the existence of confounding or “hidden” variables controlling the accumulation of organic carbon in the soil. For example, a large spread in the mean residence time in the soil as a function of molecule identity indicates that chemical structure alone is a poor predictor and that other hidden variables hold a larger deterministic role. Right: When plotted against a fully explanatory hidden variable, the spread in mean residence times would collapse onto a curve. **(E)** The existence of these deterministic quantitative relationships implies the existence of an underlying mechanism, which can be formalized into a theoretical model predicting the output (e.g., mean residence time of root biomass in the soil) as a function of an input. These models quantitatively predict the phenotype of yet unrealized genotypes. **(F)** Custom-built genotypes can then be created to systematically explore the dynamic range of the control variable and validate and further refine theoretical predictions. Along this process, genotypes with the desired engineered characteristics will be encountered.

Early studies in the 19th century showed that the rate of photosynthesis depends on temperature, light intensity, and CO_2_ concentration, which we may call the “key control variables” of photosynthesis. This was demonstrated quantitatively by Frederick Blackman and Gabrielle Matthaei and later scrutinized by Otto Warburg by progressively increasing each variable while keeping the other 2 constant [16]. By doing this, it was possible to obtain “input–output” curves where, for example, light intensity corresponds to the x-axis input and the rate of carbon fixation is the y-axis output (Fig 1A). Next, the interaction between control variables could be obtained by varying them simultaneously. These curves were shown to follow a characteristic “non-rectangular hyperbola” shape known today to every plant physiology student, with a linear increase early on that then plateaus. This simple observation indicated that, under normal light conditions, photosynthesis “saturates,” and that increments in light intensity are less and less effective at increasing photosynthesis, a fact that underlies much of the rationale behind the RIPE project engineering.

### Accounting for observations using mechanistically inspired theoretical models

Having identified the determinants of photosynthesis rate and their quantitative effects, one of the main goals of photosynthesis researchers was to come up with a way to account for these quantitative relationships. By “account” we mean here to devise mechanistically inspired mathematical formulas to describe experimental observations and predict photosynthetic output as a function of the levels of light or CO_2_ input (Fig 1B). It has to be kept in mind that the molecular basis of photosynthesis was, at best, speculative at the time, and so these models were, particularly in the beginning, hardly more than arbitrary fits to the data. For example, to account for the quantitative data from Warburg, Baly invoked a proportionality between the rate of photosynthesis, *r*, and the formation of a complex between Chlorophyll A molecules, *A*, and bicarbonate, *b*, leading to expressions such as

$$r=[Ab]=k_{1}\times \;\frac{k_{2}{\left[{CO}_{2}\right]}_{atm}}{1+k_{2}{\left[{CO}_{2}\right]}_{atm}},$$

where [*CO*_*2*_]_*atm*_ is the atmospheric CO_2_ concentration, and *k*_*1*_ and *k*_*2*_ are empirical constants. Keeping temperature and light intensity unchanged, it is perfectly possible to fit Warburg’s empirical non-rectangular hyperbola using this formula [17]. Models such as these may seem whimsically wrong to us today, but they illustrate how, from the very beginning, photosynthesis researchers engaged in a dialog between theory and experiments to hone their intuition about the problem. Although rich in arbitrary empirically derived constants and untested (and untestable at the time) assumptions, these models provided clear targets for experimental falsification and inspired the search for more mechanistic accounts for the data, gradually incorporating more molecularly grounded details. It was not until the late 1970s that a complete and more or less mechanistically accurate mathematical model of photosynthesis was developed by Farquhar and colleagues [18].

Some might argue that a complete and detailed molecular picture of all moving parts and pieces is a prerequisite for predictive understanding. The gradual evolution of mathematical models to account for the photosynthesis non-rectangular hyperbola illustrates the fact that models do not need to be comprehensive or even true to be useful. This work demonstrates how early plant physiologists were rooted in quantitative approaches that set the foundation for much of our understanding of plants. These efforts form part of a longstanding and unbroken modeling tradition in photosynthesis research, which ultimately seeks to theoretically predict rates of gas exchange at the leaf, plant, and field levels based on experimentally measured biochemical and physical constants. Today, these approaches have been assimilated into sophisticated computer simulations of crop performance (i.e., *in silico* crops) that integrate mechanistic knowledge over a wide range of scales from DNA to the field level [14,19].

### Testing model predictions using genetic perturbations

The saturating effect of light on the rate of carbon fixation implies that excess light energy absorbed by the photosynthetic apparatus must be safely dissipated. Plants have regulatory mechanisms to turn on a ‘safety valve’ under saturating light and then turn it back off when light becomes limiting again [20,21]. If the rate of these safety valve mechanisms turning off is much slower than the rate with which light fluctuates from saturating to limiting, a leaf might find itself dissipating light energy that could be diverted to photochemistry. This, in turn, could limit crop yields. The theoretical sophistication of photosynthesis as a field made it possible to put this question in rigorous mathematical terms. A model by Zhu and colleagues showed that, under fluctuating light conditions such as those normally encountered in the field, speeding the turn-off rate of this safety valve would increase yields by 20%, enough to make it an engineering target worth pursuing [20]. As further confirmation, independent theoretical studies also arrived at similar conclusions [22]. Around the time that modelers were laying out the theoretical basis for rationally engineering photosynthesis, the genetic and molecular basis for the key control variables such as the speed and degree of energy dissipation had begun to be elucidated. Fairly coarse manipulations, such as overexpressing individual genes, demonstrated that it was possible to tune the speed of energy relaxation via genetic perturbations [23] (Fig 1C). Follow-up work demonstrated that tobacco plants could be engineered to accumulate up to 15% more biomass under realistic field conditions, remarkably close to the theorized 20% [24]. Most recently, a distantly related crop species, soybean, was engineered using the same blueprint and comparable results were obtained [25]. Importantly, another group found that potato plants engineered in the same way had a reduced yield, perhaps indicating that the dialog between theory and experiments has not quite reached a conclusion yet [26] (Fig 1B and 1C).

Surely, the solid theoretical and quantitative foundations of photosynthesis research, coupled with new synthetic biology tools, promise to keep this dialog alive. Certainly, the atmosphere—governed mainly by physicochemical principles—is a dramatically simpler environment than the soil, a staggeringly complex ecosystem with solid, liquid, and gaseous phases. Given these differences, can the lessons from over a century of photosynthesis research be applied to emerging plant engineering problems such as soil carbon sequestration? We now turn our attention to answering this very question.

### Case study 2: Engineering plant-based soil carbon sequestration

Efforts to “sequester” the carbon fixed by photosynthesis are as diverse as photosynthetic organisms, ranging from the engineering of microalgae to trees. Instead of reviewing these numerous strategies, we will focus on approaches that target how carbon is introduced into soils: roots. Here, the broad stroke goal is to engineer the architecture and/or chemical composition of the root system to ultimately produce plants more likely to deposit long-lived organic carbon in soil. Conceptually, these strategies can be classified as input-centric or output-centric, depending on whether they aim to increase the rate at which carbon enters the soil or decrease the rate at which it is lost.

A proposed input-centric strategy is engineering plants with larger root systems. The rationale for this is that, all things being equal, crops that invest a larger proportion of their carbon budget into roots transfer more carbon into the soil every growth season [4,7,27]. Obviously, if all that extra carbon simply feeds more microbial respiration, the net change is zero. Hence, this strategy rests on the dynamics of carbon output, namely, on the assumption that the turnover rate of soil carbon does not increase concomitant with the increased influx of organic matter. To address this issue, one engineering goal is to change the depth profile of plant roots without necessarily changing how carbon is partitioned between above and belowground tissues. Proponents of this strategy argue that since microbial respiration decreases with soil depth, carbon from deeper roots will be longer-lived, leading to a net accumulation of soil carbon over time. Such a goal could be accomplished by, for example, rewiring auxin (Box 1) signaling to achieve a more vertical growth angle of secondary roots. Thus, just like the larger roots idea mentioned above, the effectiveness of this strategy rests on key assumptions about the dynamics of carbon release from the soil.

This leads us to output-centered plant synthetic biology strategies aimed at reducing the rate of release of soil carbon. The goal of these strategies is to increase the mean residence time of root-derived organic carbon. At their core is the assumption that different root-derived compounds can have drastically different rates of oxidation in the soil. Proponents of this approach describe some of these molecules as “recalcitrant,” implying that their degradation rates are negligible [28]. Waxes (e.g., suberin, sporopollenin, and cutins) and complex linked phenolics (e.g., lignin) are examples of such proposed molecules, which are naturally found in plant cell walls. Notably, the vast majority of the carbon in a plant is found as cell wall material, making this structure an attractive target for engineering. Accordingly, the goal of plant synthetic biologists pursuing this strategy is to increase the fraction of root carbon that is allocated to these so-called recalcitrant molecules [28]. Ultimately, exploring the validity and design space of both input-centric and output-centered approaches will be required to optimize plants for soil carbon sequestration. This could be accomplished by, for example, engineering the thickness and/or composition of the cell walls of plant roots.

Despite the attention received by these initiatives, it is not possible at present to predict with precision whether and how much organic carbon can be accumulated in the soil by growing these hypothetical genotypes. This is because, in contrast to a centuries-old field like photosynthesis, the study of soil ecology and the role of roots in it is still a young enterprise. Using the example set by photosynthesis engineering, we can then ask to what extent this young field is moving in the direction of predictive quantitative understanding.

### Control variables: Are we measuring the right thing?

There is good evidence that microbial respiration decreases with soil depth, mainly due to the lower levels of oxygen available. Consequently, it is argued that the deeper the root biomass, the more persistent its carbon will be [29–31]. Although this inverse correlation between depth and microbial respiration is well established, it remains to be determined whether and how this correlation may be affected by a change in the depth profile of plant-derived carbon. Furthermore, to advance the predictive understanding that enables sophisticated engineering, it will be necessary to formulate these relationships in quantitative terms (Fig 1D–1F). The field is certainly moving in this direction. In comparison, the role of the chemical composition of root material is much more contentious.

**Fig 2:**
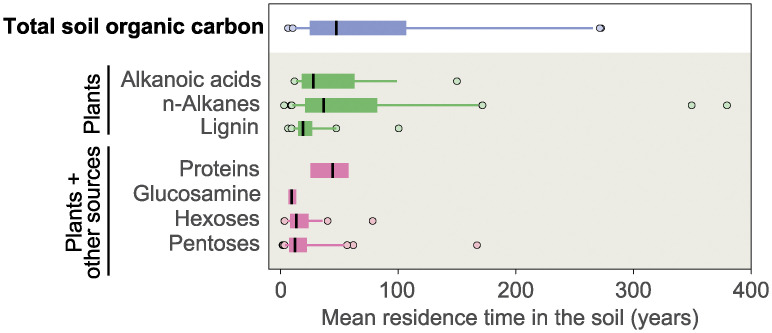
Large uncertainties in residence time of various plant-derived compounds in soil suggest major knowledge gaps and hidden variables in our understanding of soil organic carbon. Measurements of mean soil residence times reported in the literature for different kinds of organic compounds are shown (adapted from [11]). On top is the total bulk organic carbon, independent of its origin. Plant-derived compounds such as lignin, alkanoic acids, and n-alkanes have been reported to turn over in the soil in timescales ranging from months to over a century. Similar uncertainties apply to other biomolecules such as proteins and sugars whose plant or microbial origin in the soil cannot be ascertained. Central vertical lines correspond to the median, thin horizontal lines indicate the 10th and 90th percentile, horizontal bars represent the 25th and 75th percentiles, and circles indicate outliers.

A growing body of work suggests that the molecular structure of plant organic molecules does not constitute a true “control variable” determining the lifetime of soil organic matter since the same kind of molecule has widely different decay rates from experiment to experiment (Fig 1D). For example, measurements of the mean residence time of lignin in the soil can range from 6 months to a century [5,11] (Fig 2). Similar uncertainties can be found for other so-called “recalcitrant” molecules such as n-alkanes (waxes), whose mean residence time can go from 10 years to several centuries [5,11] (Fig 2). These uncertainties have sparked an ongoing paradigm shift in soil science that flips decades-old intuitive premises on their head [10]. The proponents behind this undergoing paradigm shift argue that the very fact that the degradation rate of a given molecule in the soil is so variable implies that it is not correct to consider this rate as an intrinsic property of the molecule’s structure and that other mechanisms must constitute missing variables [11]. Furthermore, they argue that the existence of long-chained recalcitrant organic molecules (also known as “humus” or “humic substances”) is nothing but an experimental artifact [8,11,32]. This is relevant, since humus was long thought to be the form that long-lasting organic carbon takes in the soil. For the purpose of plant engineering, determining the identity and magnitude of the true control variables and how and whether they interact with the molecular structure of root biomolecules is paramount. For example, some argue that it is the physicochemical properties of soil aggregates that determine the availability of organic molecules for microbial catabolism. If so, which molecules are targeted for plant engineering should depend on how they interact with a given kind of soil aggregate, rather than the structure of the target molecule itself. This is but one example of what is an active area of investigation.

### Theoretical models: Building a predictive understanding of the system

We saw that in the case of photosynthesis research, identifying the right variables and quantitatively measuring their effect was necessary but not sufficient to achieve a predictive understanding. Some sort of theoretical scaffolding was needed to account for these measurements in order to be able to predict future experiments. Making this leap will be key to guiding efforts to engineer plants to store atmospheric carbon in the soil. On a purely practical level, such predictive models would let us understand how different plant genotypes interact with the soil environment without having to engineer these genotypes or subject them to different experimental conditions, akin to how “*in silico* crops” predict the interaction of hypothetical plant genotypes with the atmosphere.

**Fig 3:**
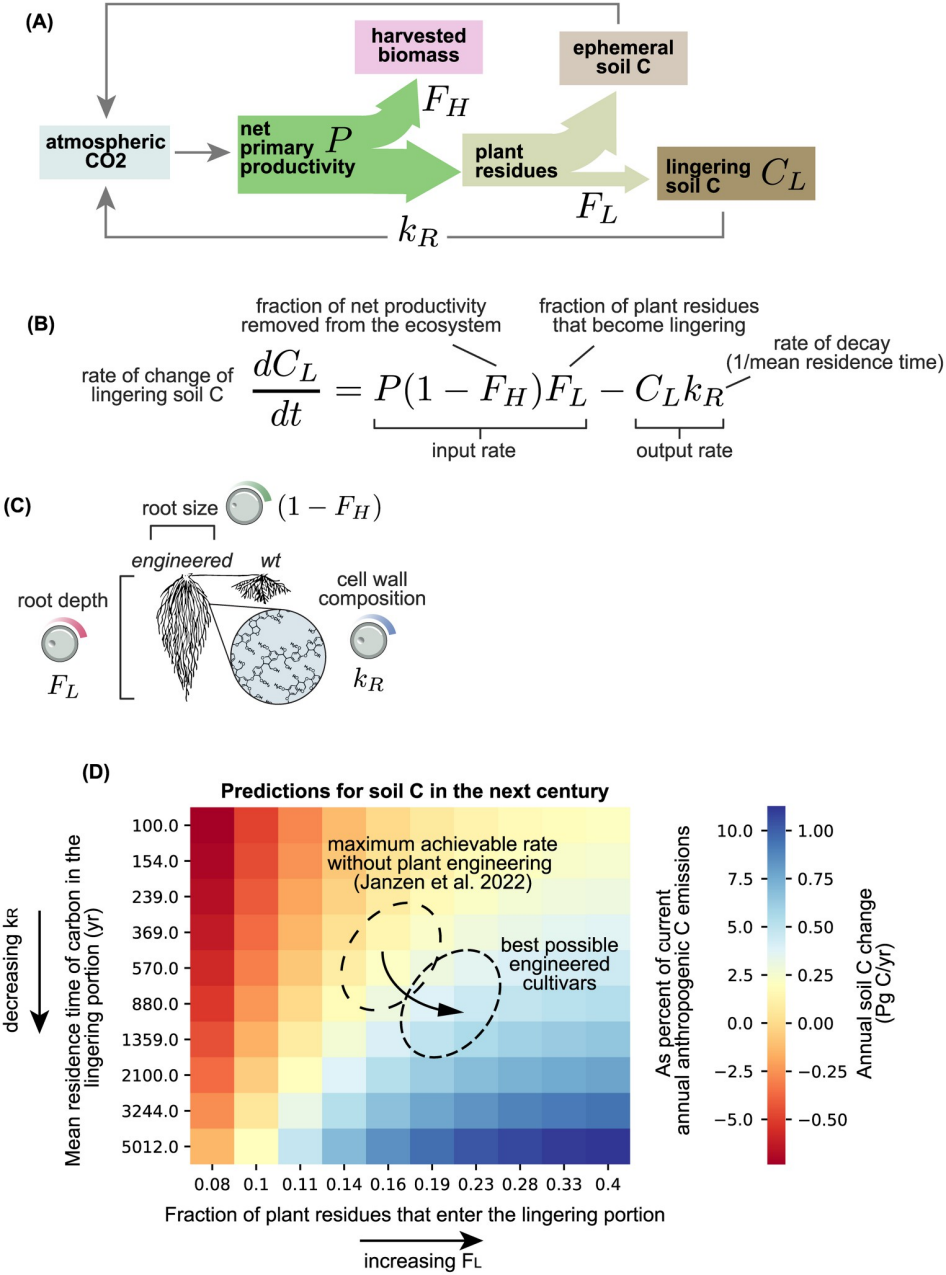
Turning mental pictures into quantitative estimates. **(A)** Cartoon capturing a common view of the fate of carbon in an ecosystem subjected to human extractive activities (e.g., cropland). Atmospheric CO_2_ enters the system through photosynthesis and is stored as net primary productivity (*P*) in plant biomass. A fraction of this biomass corresponding to *F*_*H*_ is harvested, while the rest remains in the ground as plant residues. These residues can contribute to 2 distinct pools of soil carbon, an ephemeral one with a fast decay rate (weeks to months) and a lingering pool with a much longer residence time (decades to centuries). *F*_*L*_ corresponds to the fraction of the non-harvested plant biomass that becomes part of the lingering pool. This pool can eventually be degraded back to carbon dioxide at a rate of *k*_*R*_ (where the mean residence time is 1*/k*_*R*_). **(B)** The cartoon in (A) can be made into a mathematical model describing the rate of change of the amount of carbon in the lingering pool. **(C)** Different root engineering approaches can be mapped onto specific parameters in the model in (B). For example, increasing the root size (while keeping the net productivity constant) would mean increasing the 1 *− F*_*H*_ term. Increasing the depth profile of roots would increase the *F*_*L*_ term since biomass buried deep in the soil might persist much longer. Altering the chemical composition of cell walls in roots to increase the relative abundance of so-called “recalcitrant” polymers would decrease the *k*_*R*_ term. **(D)** Using the model in (B), it is possible to predict the rate of change in soil carbon over time as a function of model parameters. Janzen and colleagues [34] used this model to predict a maximum net sequestration rate of about 0.14 Pg C yr^−1^. Combining (B) and (C), we can estimate the effect of engineering approaches. If the mean residence time (1*/k*_*R*_) of plant organic matter was engineered to increase by a factor of 2–3 times while simultaneously increasing the fraction of plant material that enters the lingering fraction (*F*_*L*_) by approximately 30%, then the net sequestration rate would be increased by about 4 times, up to 0.5 Pg C yr^−1^, or approximately 5% of the total annual anthropogenic CO_2_ emissions (assuming a global adoption of these engineering approaches).

In our first case study, we also argued that theoretical models need not be perfect to be useful. As the pharmacologist James Black put it in his Nobel address, “models in analytical pharmacology are not meant to be descriptions, pathetic descriptions, of nature; they are designed to be accurate descriptions of our pathetic thinking about nature” [33]. So far, most models of soil carbon dynamics have been of the first kind, attempting to accurately account for all available knowledge. As a consequence, they can be so intractably complex that they can hardly be falsified in a controlled laboratory setting and add little in terms of sharpening our understanding of the problem. One way to build an “accurate description of our pathetic thinking” is to turn the way we talk about a problem into a mathematical equation. This was recently attempted by Janzen and colleagues who took the commonly offered rationale for plant-based soil carbon sequestration as we described it above and reformulated it in mathematical terms [34] (Fig 3A). The result is an equation predicting the rate at which the carbon in the soil accumulates over time. This rate is the result of an input rate consisting of the fraction of net primary productivity that gets stashed away in a lingering, long-lived pool minus an output rate governed by the rate of decay of carbon in this pool (Fig 3A and 3B). Similar approaches are commonly used to describe agronomically relevant traits, such as harvest index (Box 1) [35], demonstrating how it is possible to formalize specific parameters to estimate their impact on crops. For example, proposed changes to the molecular composition of plant roots would decrease the *k*_*R*_ term (the rate of decay of the lingering carbon pool) while engineering deeper root systems would increase the *F*_*L*_ term (the fraction of plant residues entering the lingering pool) (Fig 3C). This example captures the kind of intuitive yet falsifiable models reminiscent of early photosynthesis equations and could serve as a starting point for the field. In addition, they enable educated guesses to be made about the impact of competing engineering solutions in order to make decisions (Fig 3D).

### Identifying control variables and testing theoretical predictions: The role of synthetic biology

Unlike the case of photosynthesis, where the control variables can be easily manipulated by the experimenter, the mechanisms that are thought to dictate the fate of root carbon in the soil are much less tractable. In many cases, it is not even clear how one would obtain something analogous to the input–output curves that we described in the case of photosynthesis. Many of these control variables incorporate the fate of carbon through soil microbes or take into account the physico-chemical properties of the soil, illustrating that what we have covered here is only a slice of a much more complicated story. Entire subfields are emerging from such questions, such as exploring how to rationally engineer the soil microbiome. Still, many control variables are genetically encoded by plant genomes; however, due to the lack of precise perturbation tools, the study of these candidate mechanisms has remained at an observational level. Synthetic biology approaches could be leveraged to genetically engineer plants to systematically test these molecular determinants in a controlled laboratory setting. To illustrate this concept, we now give 3 specific examples, which are by no means exhaustive.

The first potential hidden variable is the composition of root exudates [36,37]. It has been argued that the simple and highly reduced carbohydrates found in some exudates can provide the energy necessary to prime microbial metabolism. Once primed in this way, microbes are capable of feeding on practically any biomolecule, “recalcitrant” or otherwise. Using plant metabolic engineering, it may be possible to specifically alter the composition and profile of these exudates while keeping their amount and physical properties equivalent, and in this way answer whether the availability of simple energy-rich sugars in the soil constitutes a hidden variable (Fig 1D). If this is the case, the same synthetic biology tools could be used to systematically explore the dynamic range of this variable to test predictive models linking the amount and composition of root exudates to the catabolic activity of soil microbes (Fig 1D–1F).

A second possible variable is the role of the cell wall ultrastructure in controlling the access of microbial catabolism to cell wall polymers rather than the polymer chemical composition itself. For example, the thread-like fibers formed by cellulose make it one of the hardest plant materials for microorganisms to break down, even though chemically it is one of the simplest plant polysaccharides (a chain of β(1→4) linked glucose). Hence, instead of focusing on engineering the molecular composition of root cell walls as if they were a bag of chemicals, the target of manipulations would be the cell wall ultrastructure, meaning how these molecules are bound, cross-linked and arranged with respect to each other in the cell wall matrix. This is an example of a hypothesis driving the need for new synthetic biology tools, since there are currently no methods to modulate the plant cell wall ultrastructure.

Finally, the hidden variable that is perhaps the most within reach is the depth profile of plant roots. Using synthetic biology tools, it is possible to introduce constructs into a single genetic background that confer a varying degree of root depth [38,39]. At the same time, there has been progress in measuring and predicting the rate of microbial respiration as a function of depth into the soil profile. Hence, it should be possible to combine these 2 areas to advance theoretical predictions of the residence time of root carbon as a function of root depth.

These predictions could then be experimentally tested using custom-built plant genotypes.

### Conclusions and future outlook

It took about a hundred years to go from a quantitative description of the determinants of photosynthesis rate to current rational engineering approaches based on computer simulations. Unfortunately, the imminent consequences of climate change necessitate our ability to more rapidly deliver solutions in mitigating anthropogenic greenhouse gas emissions. We have chosen to focus on the history of the rational engineering of photosynthesis because it is immediately familiar to plant biologists, but the blueprint we laid out would probably be recognizable to students of engineering in any field. With the benefit of hindsight, photosynthesis might seem to be a comparatively simpler problem than that of soil carbon ecology. It likely did not seem so to the generations of researchers whose efforts resulted in modern day models of plant photosynthesis containing hundreds of painstakingly obtained parameters, ranging from protein concentrations and enzymatic constants to gas diffusion rates. Yet, the overwhelming goal of engineering the pumping of inorganic carbon from the atmosphere into the soil via plants is undeniably much larger in scope. This realization is humbling and underscores the importance of nailing down the underlying principles to guide not just engineering efforts, but our basic understanding in the coming decades of this relatively young field.

The urgency of ameliorating the worst effects of raising atmospheric CO_2_ means that we cannot afford hastily “throwing ideas to the wall and seeing what sticks.” As we highlighted in our second case study, there is, to date, no agreement of what the molecular determinants that promote accumulation of organic matter in the soil are. By not immediately addressing these key knowledge gaps, engineering soil carbon sequestration is doomed to remain exploratory. Fortunately, there are abundant falsifiable hypotheses ripe for the picking, if only we had the right molecular tools to test them. We have provided some examples of how novel plant synthetic biology approaches could be leveraged to this end. If such technologies were tunable, it would then be possible to obtain quantitative input–output relationships, the prerequisite for building a predictive understanding according to our first case study.

Finally, we wish to emphasize that, ultimately, the examples we have chosen for our case studies are arbitrary illustrations of a larger principle, namely, that future plant engineering solutions will be the natural byproduct of the push and pull between theoretical predictions and the synthetic genotypes designed to test them.
